# Supplementary material for: Chromothripsis during telomere crisis is independent of NHEJ, and consistent with a replicative origin
Source: Genome Res. 2019 May;29(5):737–49. doi: 10.1101/gr.240705.118 (PMC6499312; doi:10.1101/gr.240705.118)
Supplement: Supplemental Material [file supp_gr.240705.118_Supplemental_file_1.zip › contigs/annotated_contigs/DB86/contig.2.DB86_length_311_mean_cov_4.34083601286.docx]

**DB86_length_311_mean_cov_4.34083601286**

```
TGAATCTATATTTTACCTTAACTTTTATTTATTTTTATTTTTTAAATTTTATTTTATGAATATCTAATCTGCCAGCATTTTAGAAATTT
 >chr1:57072958-57073164 - E=8e-113 p=9e-03
GTTTCTATTTTCTAAATTTTATTTTATGATACTTTCACTTTTAAAGGATATTTTTTCTGGGTATATAGTTCCAGATTGACAGTTTCTGT

CCCATTTCAATACTTTATGTTGTT|ACAT|AAGATTTTTCTTTAGTTAGAACTCAGTTACATAGAAATGTAGCAATCATGCTTGGTTGA
                         >chr1:57153289-57153398 - E=3e-54
TGACACTTGTCTTCAGTAGTCCATGAATGACCTTTTATTTAATTAC
```
